# Cardiac tumor in the left ventricular outflow tract: atypical presentation of calcified amorphous tumor

**DOI:** 10.1093/jscr/rjac179

**Published:** 2022-05-18

**Authors:** Toyokazu Endo, Kachon Lei, Chowdhury Ahsan

**Affiliations:** Department of Internal Medicine, Kirk Kerkorian School of Medicine at UNLV, Las Vegas, NV USA; Department of Internal Medicine, Kirk Kerkorian School of Medicine at UNLV, Las Vegas, NV USA; Division of Cardiovascular Medicine, Kirk Kerkorian School of Medicine at UNLV, Las Vegas, NV, USA; Division of Cardiovascular Medicine, Kirk Kerkorian School of Medicine at UNLV, Las Vegas, NV, USA

## Abstract

Calcified amorphous tumor (CAT) of the heart is a rare nonneoplastic tumor. A 71-year-old woman presented with a mobile mass within the left ventricular outflow tract during her elective transthoracic echocardiogram. The patient exhibited no symptoms, and intraoperative findings showed the mass originating from the anterior leaflet of the mitral valve. Transthoracic and transesophageal echo failed to diagnose and localize the origin of the tumor. The tumor origin is unclear, but CAT of the mitral valve may be associated with mitral annular calcification. Surgical excision of the mass definitively diagnoses the tumor and reduces the risk of embolization.

## INTRODUCTION

Calcified amorphous tumor (CAT) of the heart is a nonneoplastic tumor that can originate from any of the four chambers. It was first described in 1997, and since then, only a few cases have been reported in the literature [1]. The most recent systemic review of the literature indicated that these tumors are common in females and found on the mitral valve 36% of the time. Most patients do report symptoms, but some exhibited none [2]. Here, we present a unique presentation of CAT that was incidentally found in the left ventricular outflow tract (LVOT) on transthoracic echo but intraoperatively, the mass originated from the mitral valve leaflet.

## CASE REPORT

A 71-year-old woman was hospitalized for an elective LVOT tumor removal. The mass was incidentally discovered on her elective transthoracic echocardiography (TTE) during her outpatient cardiology visit. She had a history of non-insulin-dependent diabetes and 11 year history of multiple myeloma in remission. Her medications included lenalidomide and pomalidomide. Prior to the echo, the patient exhibited no symptoms, including dyspnea on exertion, syncope, angina or embolic events. No other cardiac workup was performed prior to this echo.

TTE showed a mobile nonobstructive mass within the LVOT that was 0.4 cm in size. The aortic valve was tri-leaflet with mild sclerosis without stenosis. Also, the mitral valve had moderate calcification on the leaflets with both mild stenosis and regurgitation. A left heart catheterization revealed no coronary artery obstruction from the mobile mass at the level of the sinus of Valsalva. The patient was scheduled for elective removal of the tumor due to the risk of embolization, which is often a complication of CAT and cardiac fibroelastoma. Preoperative physical exam and electrocardiogram were unremarkable. Her pre-op labs were only remarkable for an elevated calcium level of 10.3 mg/dl, which was attributed to her diagnosis of multiple myeloma.

**Figure 1:**
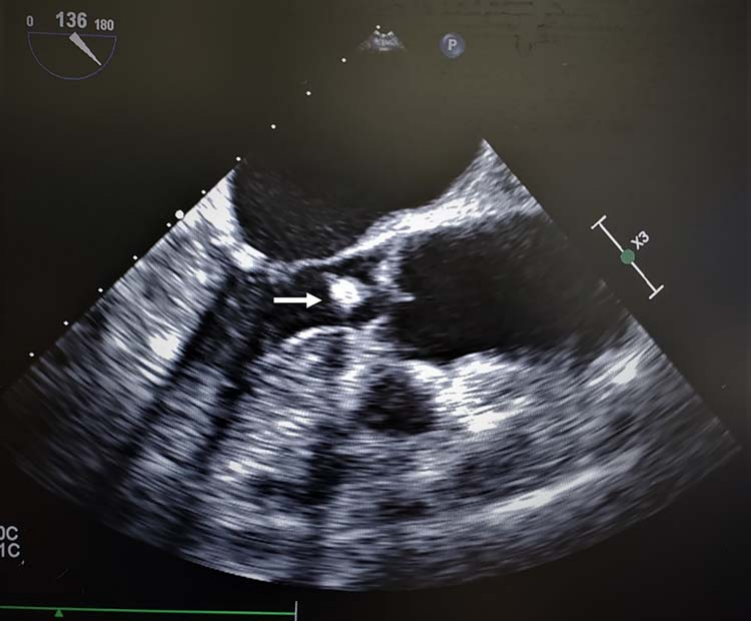
Transesophageal echocardiogram of cardiac amorphous tumor within the LVOT; intraoperative TEE revealed similar findings as a TTE in the outpatient setting; the hyperechoic sphere-like lesion can be identified within the LVOT in the mid-esophageal bicaval view (white arrow); the mass was mobile and nonobstructive to the LVOT; the attachment site was not visualized clearly.

Intraoperative transesophageal echo (TEE) revealed similar findings as to the TTE (Fig. 1). The mass appeared to be pedunculated and appeared to be associated with the noncoronary cusp of the aortic valve. The hyperechoic sphere-like mass moved with each ventricular contraction. The mass moved across the aortic valve into the sinus of Valsalva without evidence of aortic insufficiency.

**Figure 2:**
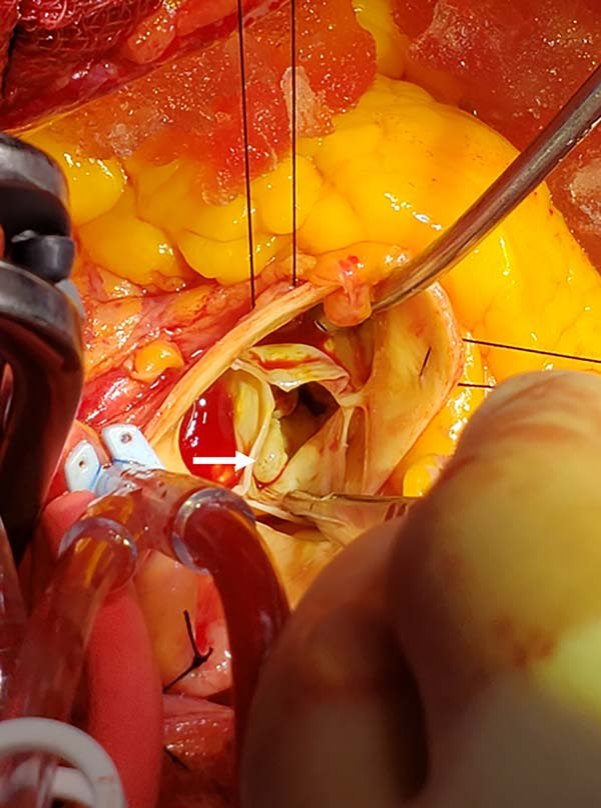
Sphere-like mass identified under the noncoronary cusp of the aortic valve; intraoperatively, the mass was under the noncoronary cusp of the aortic valve; the mass was traced and was originating from the A1 segment of the mitral valve.

The initial gross inspection of the aortic valve showed all three cusps without sclerosis. Under the aortic valve, the sphere-like mass was identified (Fig. 2). Contrary to TTE and TEE findings, the mass was elongated along the LVOT and was attached to the anterior leaflet (the A1 segment) of the mitral valve. The mass was resected, and mitral valve repair was not indicated. The patient did require a pacing wire for few days due to atrioventricular nodal disassociation but recovered well without prolonged complications.

**Figure 3:**
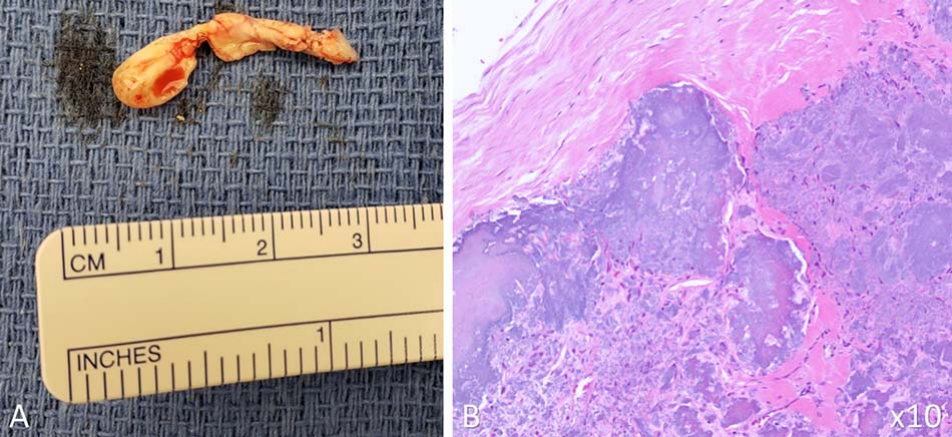
Gross picture and histological slide of CAT; the mass was isolated in two irregular fragments with the following dimensions: 5 × 4 × 3 mm and 22 × 7 × 6 mm (**A**); histological examination of the mass under 10x magnification (**B**) (hemolysin and eosin staining) showed nodular calcification in the background of eosinophilic amorphous background; both gross and histological findings were consistent with CAT of the heart.

The gross specimen of the tumor was firm with apparent calcification with tan to gray appearance (Fig. 3A). Microscopic examination revealed cardiac valve tissue with nodular calcification and mild focal acute inflammation. The gross specimen and the histological description were consistent with CAT of the mitral valve (Fig. 3B).

## DISCUSSION

CAT is a rare heart lesion, and only a handful of cases have been reported. The exact origin of CAT remains unknown. From the most recent systemic review published in 2015, these tumors have multiple different presentations [2]. However, there appears to be some pattern of CAT that is associated with mitral valves or annulus. A total of 13 of the 42 cases had CAT on the mitral valve or the mitral annulus (MA). CAT associated with mitral valve was more likely to present with systemic embolic events. Additionally, those with CAT on MA were more likely to have associated mitral annular calcification (MAC). Like CAT, the etiology of MAC remains unknown [4]; however, CAT of the MA may be linked to the process involved in MAC given its calcific nature of the tumor. Future directions should focus on the exact origin of CAT and understand the best approach to prevent adverse pulmonic or systemic embolic events.

Although there has been a case reporting a CAT on the MA extending to the closing aortic valve, our case is unusual due to its unclear location of tumor origin via left heart cath or Echo [3]. This case highlights the current limitation of diagnosing cardiac lesions accurately, and cardiac surgery remains a crucial step in the actual diagnosis and treatment of CAT.
